# *Leuconostoc gelidum* Is the Major Species Responsible for the Spoilage of Cooked Sausage Packaged in a Modified Atmosphere, and Hop Extract Is the Best Inhibitor Tested

**DOI:** 10.3390/microorganisms12061175

**Published:** 2024-06-10

**Authors:** Giuseppe Comi, Andrea Colautti, Cristian Edoardo Maria Bernardi, Simone Stella, Elisabetta Orecchia, Francesca Coppola, Lucilla Iacumin

**Affiliations:** 1 Department of Agricultural, Food, Environmental and Animal Science, University of Udine, Via Sondrio 2/a, 33100 Udine, Italy; andrea.colautti@uniud.it (A.C.); orecchia.elisabetta@spes.uniud.it (E.O.); lucilla.iacumin@uniud.it (L.I.); 2 Department of Veterinary Medicine and Animal Sciences, University of Milan, Via dell’Università 6, 26900 Lodi, Italy; cristian.bernardi@unimi.it (C.E.M.B.); simone.stella@unimi.it (S.S.); 3 Food Sciences Institute, National Research Council, Via Roma, 64, 83100 Avellino, Italy; fracop93@gmail.com

**Keywords:** cooked sausage, spoilage, *Leuconostoc gelidum*, volatile compounds, preservation agents

## Abstract

Cooked sausages packaged in a modified atmosphere (MAP: 20% CO_2,_ 70% N_2_, <0.2% O_2_) with evident yellow stains were analyzed. The aims of this work were to study the microbial cause of the spoilage and to evaluate different antimicrobial compounds to prevent it. *Leuconostoc gelidum* was identified as the primary cause of the yellow coating in spoiled cooked sausage, as confirmed by its intentional inoculation on slices of unspoiled sausage. *Leuconostoc gelidum* was the main bacteria responsible for the yellow coating in spoiled cooked sausage, as confirmed by its intentional inoculation on slices of unspoiled sausage. The yellow color was also evident during growth in the model system containing cooked sausage extract, but the colonies on MRS agar appeared white, demonstrating that the food substrate stimulated the production of the yellow pigment. The spoilage was also characterized by different volatile compounds, including ketones, ethanol, acetic acid, and ethyl acetate, found in the spoiled cooked sausage packages. These compounds explained the activity of *Leuc. gelidum* because they are typical of heterofermentative LAB, cultivated either on food substrates or in artificial broths. *Leuc. gelidum* also produced slight swelling in the spoiled packages. The efficacy of different antimicrobials was assessed in model systems composed of cooked sausage extract with the antimicrobials added at food product concentrations. The data showed that sodium lactate, sodium acetate, and a combination of sodium lactate and sodium diacetate could only slow the growth of the spoiler—they could not stop it from occurring. Conversely, hop extract inhibited *Leuc. gelidum*, showing a minimal inhibitory concentration (MIC) of approximately 0.008 mg CAE/mL in synthetic broth and 4 mg CAE/kg in cooked sausage slices. Adding hop extract at the MIC did not allow *Leuc. gelidum* growth and did not change the sensorial characteristics of the cooked sausages. To our knowledge, this is the first report of the antimicrobial activities of hop extracts against *Leuc. gelidum* either in vitro or in vivo.

## 1. Introduction

Retailers sell cooked delicatessen products as pre-sliced meats that are packaged in a modified atmosphere (MAP: 30% CO_2_, 70% N_2_, 0.2% O_2_). Their shelf life is fundamentally influenced by the microbiota present. The actual microbial contamination occurs during the packaging process because very few bacteria are able to withstand the heat treatments required for cooking or pasteurization (postcooking) [1]. Moreover, microorganisms enter the product when the wrapping is removed before slicing, when the slicing is complete, and when it is packaged again. At best, using a clean room can lessen contamination; it never totally eradicates it. In fact, plant hygiene—including food handlers adhering to personal hygiene standards—is extremely important [1,2]. The shelf life could also be impacted by packaging-related secondary contamination, in addition to the microbiota introduced through heat treatment [1]. Additionally, a significant factor that affects the outcomes of the bacterial analysis of both raw and cooked sausage is the incubation temperature. Different kinds of changes are produced during the processing of such products [3,4]. After cooking, various authors have tracked the contamination of food items and noted that it can occur at up to 5 log CFU/g [2].

Cooked cured meats that are sliced and packaged in MAP typically have a 21–30 day shelf life, but they can last up to 45 days [3]. To gain a competitive edge in the market, manufacturers work hard to provide products with the longest shelf lives [1]. Strong acidification, volatile organic compounds, the development of colored films, unpleasant molecules, and other changes typical of spoiled packaged food are produced by microbial metabolism [5]. Above all, lactic acid bacteria (LAB), especially psychrotrophic strains that can grow at lower temperatures than mesophilic strains, are the cause [6]. In actuality, as previously recommended by Dušková et al. [7] and Pothakos et al. [8], selective media must be used in conjunction with varying temperatures for a thorough assessment of the presence of LAB in cooked delicatessen goods. Therefore, when assessing the amount of spoilage bacteria in products kept under refrigeration, an incubation temperature of 30 °C does not provide an entirely impartial image of the microbiota present. Therefore, it is crucial to establish the ideal cultivation temperature to produce a representative sample of LAB found in meat products [7,8]. The primary bacteria that cause the deterioration of cooked and vacuum-packed meat products are LAB strains [9]. *Latilactobacillus sakei* and *Latilactobacillus curvatus* are frequently recovered from cooked meats that have been refrigerated; *Lactobacillus* spp., *Leuconostoc* spp., and *Weissella* spp. are the predominant genera linked to the spoilage of these products [7,10,11].

Spoilage can occur during the shelf life period due to an increase in the level of contamination by LAB or particularly active spoilage strains, which causes the manufacturer to repeatedly recall the product [9,11]. Based on the evaluation of the initial microbial load and the spoiled product, studies to ascertain the causes of variations in the quality of cooked and pre-sliced products [9] revealed that LAB contamination occurred after cooking and was mediated by air, which picked up the microorganisms from the macerated raw meat and transferred them to the cooked product. Specifically, the modification and sensory qualities of the pre-sliced products were more significantly impacted by LAB derived from raw beef in brine [9]. A significant increase in the isolation of psychrotrophic and mesophilic *Leuconostoc* strains was noted in MAP cooked ham. In particular, psychrotrophic *Leuconostoc* is found in food products that are not supposed to be consumed due to spoilage. These products include concentrations that, when found in excess of 10^7^ CFU/g, have been observed to produce strange colors, slime, disagreeable odors, and discoloration [6,10,11,12,13,14]. The ability of *Leuconostoc* spp. to grow psychrotrophically and proliferate at high CO_2_ concentrations is the main reason they are more widespread than other LAB species [5]. Some species in the genus *Leuconostoc* are economically significant because of their positive or negative effects on specific food preparations [14]. They have negative effects on food fermentation (e.g., sauerkraut, salami, and meat products) and positive effects by the creation of aromatic compounds in a variety of dairy products [1,5], the on-site synthesis of dextran in dairy products that contain sucrose, and the synthesis of functional molecules [11,15]

Since they are also the source of rancidity, strange scents, coloring (greening), stickiness owing to exopolysaccharide (EPS) synthesis, and gas (CO_2_) production with food package enlargement, their specific features allow them to modify the ecosystems of meat and dairy products [12,15,16].

Since there have been no reports of spoiled cooked salami in the literature, this study’s goal was to determine which LAB was responsible for the observed spoilage. Specifically, the spoilage of a batch of cooked salami packaged in MAP with a shelf life of approximately 1 month at 4 °C was studied. Additionally, we aimed to find the influence of traditional or unconventional (hop extract) antimicrobial compounds to inhibit spoilage. Among the antimicrobial compounds against pathogenic and spoilage microorganisms, the scientific literature suggests using hop extract. Indeed, hop extracts have long been known to have antimicrobial activity [17,18,19,20]. However, most studies have evaluated their activities in culture media or in wort and beer but not in food [17]. Considering that hop extracts inhibit Gram-positive bacteria, including species of *Bacillus*, *Micrococcus*, *Staphylococcus*, and others, we wanted to investigate the antimicrobial effects against the spoilage microorganisms isolated in these spoiled cooked sausages.

## 2. Materials and Methods

### 2.1. Selected Samples and Sampling Procedures

**Figure 1:**
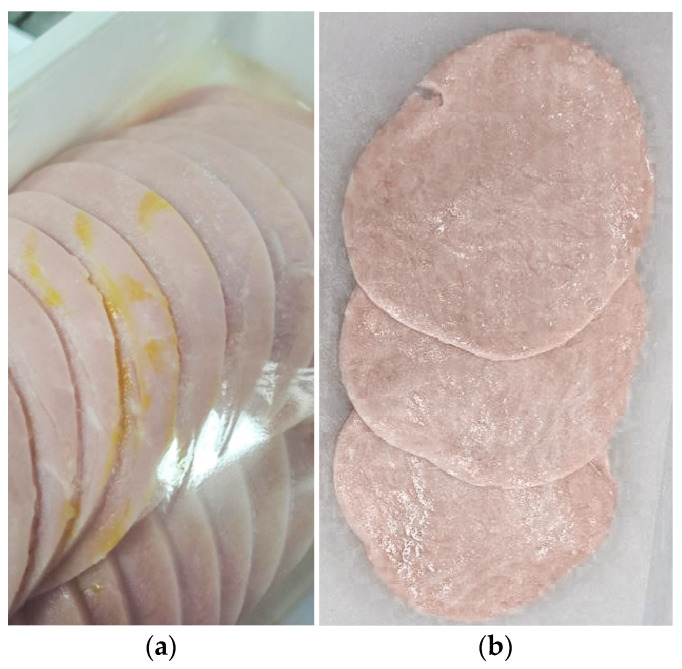
Growth of *Leuconostoc gelidum* on cooked sausage: (**a**) growth; (**b**) no growth.

From a batch consisting of 200 packs of cooked sausage produced by an artisan from Friuli, approximately 160 samples were selected with evident spoilage presence (Figure 1a). The sausages were produced using the flow sheet shown in Table 1, with the following ingredients: pork meat, pork belly, pork rind, salt, dextrose, sucrose, flavorings, and spices. Antioxidants: E301-E331. Preservative: E250. After production and slicing, the cooked sausages were packaged in trays made of polypropylene PP, while the sealing film was 50-micron-thick PE/PP polyethylene/polypropylene in MAP (30% CO_2_, 70% N_2_, and <0.3% O_2_) using a packaging machine (Orved—VM—53 Italy) and stored at 4 ± 2 °C. Within 10 days of storage, 160 samples were spoiled and 40 unspoiled. The spoilage consisted of a yellow patina (slime); in some cases, the packages were slightly swollen.

A total of 70 spoiled and 30 unspoiled packages were visually inspected and analyzed through chemical–physical and microbiological methods. Furthermore, 10 packages with or without slime present were selected and evaluated for volatile compounds.

### 2.2. Microbiological Analyses

From each package, all of the slices with or without slime were placed in Stomacher bags, diluted in sterile peptone water (0.1 g/L peptone, 7 g/L NaCl), and homogenized in a Stomacher (P.B.International, Italy). Then, the mixture was diluted with the same solution, and 1 mL of each dilution was placed in Petri dishes, to which different growth media were added. The analyses were performed in triplicate and included a search for total aerobic microorganisms in plate count agar (PCA, Oxoid, Italy) incubated at 30 °C for 48 h (ISO 6887), lactic acid bacteria (LAB) in De Man–Rogosa–Sharpe (MRS) agar (pH 6.2, Oxoid, Italy) incubated at 25 °C for 48–72 h (ISO 15214) in jar with gas pack anaerobic system (BBL, Becton Dickinson, Milpitas, CA, USA).

From the MRS plates, which contained 30 to 300 colonies, 600 colonies were randomly isolated. These were selected regardless of morphology, color, and size. The isolated colonies were purified on MRS agar and then stored at −80 °C in MRS broth supplemented with glycerol (30% Sigma-Aldrich, Schnelldorf, Germany). They were then subjected to Gram staining and catalase testing and were identified according to the methods (PCR-DGGE and sequencing) reported by Iacumin et al. [16]. In particular, the DNA was amplified with the primers P1V1GC (GC-GCGGCGTGCCTAATACATGC) and P2V1 (TTCCCCACGCGTTACTCACC) [21,22]. The PCR products were processed via DGGE, and the isolates were grouped according to their migration profile. At least 3 strains from each group were subjected to sequencing for identification purposes. Furthermore, a culture-independent method was also used. Briefly, 10 mL of each dilution was centrifuged at 12,000 rpm, and the pellet was subjected to DNA extraction. The extracted DNAs were treated by PCR-DGGE [16]. Thirty bands migrating the same position in every single gel were excised, cloned, and sequenced [16]. Sequence comparisons were performed in GenBank using the Blast program version 2.2.18 (https://blast.ncbi.nlm.nih.gov/Blast.cgi; accessed on 22 February 2024) [23].

### 2.3. Chemical–Physical Analyses and Identification of Volatile Compounds

The pH was evaluated using a pH meter (Radiometer, Copenhagen, Denmark). Volatile compounds were identified by SPME-GC-MS on a Finnigan Trace DSQ (Thermo Scientific Corporation, USA) with an Rtx-Wax capillary column (length 30 m × 0.25 mm id., film thickness 0.25 µm, Restek Corporation, Waltham, MA, USA) according to the method reported in Chiesa et al. [24]. The volatile compounds were identified by comparing the spectra obtained with the spectra available in the Commercial Wiley library and from an internal library. The results are expressed as the average of 10 samples analyzed in triplicate.

### 2.4. Inhibitory Activity of Sodium Lactate (SL), Sodium Diacetate (SLD), and Sodium Acetate (SD) in a Cooked Salami Model System

The medium representing the model system was obtained by boiling a homogenate of 1.5 kg of salami in 6 l of distilled water (ratio 1/4). The mixture was boiled for 1 h, cooled to 4 °C, and filtered through Whatman 1 paper. The filtrate, which represented the model system, was divided into 50 mL flasks. Antimicrobials were added to the flasks (Table 2). Controls without antimicrobials were also made.

The flasks were inoculated with a suspension of *Leuc. gelidum* at a final concentration of approximately 2 log CFU/mL. The inoculum consisted of a mixture of 3 different strains of *Leuc. gelidum* isolated from the spoiled cooked sausages. In short, from single colonies of the 3 strains grown at 25 °C in MRS agar after 48 h, a loopful was taken and diluted in peptone water until an O.D. of 0.1 at 600 nm was reached. The concentration of the individual suspensions was determined by sequential dilutions in MRS agar and was at a level of 7 log CFU/g. Then, the suspensions were mixed and diluted until a concentration of log 4 CFU/mL was reached, which represented the mother suspension. Half a milliliter of each suspension was inoculated into flasks containing the model system media. After the inoculum, a group of samples was stored at 4 °C for up to 30 days, and a second group at 4 °C for 10 days (1/3 shelf life) and then at 8 °C (thermal abuse) for the remaining 20 days (2/3 shelf life). Both groups were analyzed at 0, 5, 10, 20, and 30 days to monitor the growth of *Leuc. gelidum* in the presence of antimicrobial agents. At the indicated times, aliquots of the media were diluted in peptone water, and 1 mL of each aliquot was analyzed through the bilayer method using MRS agar media and incubated for 48 h at 25 °C. The analysis was performed in triplicate for each time point and each antimicrobial concentration.

### 2.5. In Vitro Evaluation of the Phenotypic Characteristics of Both the Isolated Strains

The evaluation of the pasteurization effect (test 1), the value of the generation time (GT) in hours (test 2), and the pH evolution (test 3) were made using the cooked salami extract and suspensions, as reported in Section 2.4. An inoculum consisting of a mixture of 3 different strains of *L. sakei* isolated from the spoiled cooked sausages was produced by the same method for *Leuc. gelidum* and used for the three tests (Section 2.4).

Test 1: Pasteurization effect: The media were inoculated with a suspension of the identified species of approximately 6 log CFU/mL. The media were treated at 74 °C for 5 min, and after cooling, they were analyzed to identify surviving microorganisms (test 1). One milliliter of each suspension was inoculated in MRS agar using the double-layer technique, and the plates were incubated at 25 °C for 48 h.

Test 2: The generation time was evaluated by inoculating 1 mL (2 log CFU/mL) of the suspensions obtained (see Section 2.4) into flasks with cooked salami extract, and the flasks were incubated at 4 °C. At 7 days (168 h), 1 mL of each broth culture was analyzed as previously described in test 1.

Test 3: The pH achieved was also evaluated using the method reported in Section 2.3. For all the tests, 10 replicates were performed.

### 2.6. Hop Extract Preparation, Minimum Inhibitory Concentration (MIC), and Antimicrobial Effects

The dried cones were homogenized with a mortar and pestle to a fine powder. The extract was prepared using ca. 2 g of the homogenized sample and 20 mL of 96% ethanol. The extractions were carried out at 60 °C for 24 h in a water bath with constant mixing at 170 rpm [25]. The cooled extracts were then centrifuged at 2500× *g* for 10 min, and the resulting supernatants were filtered through Whatman 1 paper and stored at −20 °C until analysis. The total phenolic content was determined by the reduction of phosphotungstic acid and phosphomolybdic acid (i.e., the Folin–Ciocalteu agent) to blue pigments, and the phenolic content in alkaline solutions was determined according to the methods of Singleton and Rossi [26]. The supernatant was analyzed in triplicate, and the total phenolics were expressed as equivalents of chlorogenic acid (CAE) in mg per gram dry sample.

The MIC for the hop extract was determined in MRS (Oxoid, Italy) broth. Several single cultures of *Leuc. gelidum* strains, which were randomly isolated from cooked sausage and grown for 3 days at 25 °C on MRS agar (Oxoid, Italy), were removed and diluted in peptone water until an O.D. of 0.1 at 600 nm was reached. To evaluate the concentration of each suspension, equivalent dilutions were prepared using sterile peptone water, and 0.1 mL of each dilution was surface cultured on MRS agar plates. The plates were incubated at 25 °C for 3 days in jar with gas pack anaerobic system (BBL, Becton Dickinson, USA), and the resulting colonies were counted. Each suspension contained approximately 7 log CFU/mL. Then, the suspensions were diluted and added to the broth containing the hop extract at final concentration of 2 log CFU/mL. The antimicrobial effects were evaluated by adding 1 mL of hop extract to 9 mL of MRS broth inoculated with the suspension (at a final concentration of 2 log CFU/mL). The final CAE concentrations in the broth were 0.08 mg of CAE/mL, 0.04 mg of CAE/mL, 0.016 mg of CAE/mL, 0.008 mg of CAE/mL, and 0.004 mg of CAE/mL. The control samples were prepared by adding 1 mL of ethanol without hop extract in 9 mL of MRS broth and inoculated with the bacterial suspension. After incubation for 30 days at 25 °C, the MICs were determined as the lowest concentrations where no viability was observed on the basis of a lack of metabolic activity. The MIC measurements were carried out in triplicate.

### 2.7. Effects of Hop Extract on Cooked Sausage Slices (MICs)

One hundred slices of cooked sausages were packaged under vacuum in trays made of PP polypropylene, while the sealing film was PE/PP polyethylene/polypropylene, 50 microns thick, and they were pasteurized at 85 °C for 15 min. Then, they were unpackaged and added with hop extract solution (at final concentrations of 0, 2, 3, 4, or 5 mg CAE/kg) and inoculated with a suspension obtained by mixing the three single *Leuc. gelidum* suspensions (2 log CFU/g) to determine the MICs. The samples were packaged in MAP (30% CO_2_, 70% N_2_, and <0.3% O_2_) and stored at 4 °C for up to 30 days and at 4 °C for 10 days (1/3 shelf life) or 8 °C (thermal abuse) for the remaining 20 days (2/3 shelf life). Control samples were also made using an ethanol solution without hop extract. Five samples for each hop extract concentration and temperature were used.

### 2.8. Sensory Analysis

To evaluate the influence of the hop extract treatment on the organoleptic characteristics of the cooked sausage slices stored under vacuum at 4 ± 2 °C for 30 d, the triangle test methodology ISO 4120:2004 [27] was used. The samples included either the hop ethanol extract (4 mg of CAE/kg of product) or no extract as a negative control. Only the CAE concentration representing the MIC was used. The treated and untreated samples were compared. In brief, at 30 days, the different samples from each treatment (with or without hop extract) were subjected to the triangle test. A total of 20 nonprofessional (10 female, 10 male; average age, 30 years old) subjects, representing real consumers, were involved in the sensory evaluation. Three samples, coded with three-digit numbers, were given in randomized service order, and the assessors were asked to find out the different ones. Sliced cooked sausages were presented, wrapped in aluminum foil, in a quiet room, and the answers were collected on a paper card. Statistical evaluation of the results was carried out according to Stone and Sidel [28].

### 2.9. Statistical Analysis

Statistical testing was carried out using the specific software Statistica for Windows, version 8.0 (StatSoft, Tulsa, OK, USA). Means and standard deviations were calculated, and the data were analyzed via principal component analysis (PCA), factorial ANOVA (two factors, starter culture and time), and Tukey’s HSD test. Significant differences among the samples were defined as those for which *p* < 0.05.

## 3. Results and Discussion

### 3.1. Identification and Phenotypic Characterization of the Isolated Strains

Both LAB and non-LAB bacteria were analyzed in spoiled and unspoiled cooked sausages. The level of non-LAB was always less than 10 CFU/g products (which is the lower limit of determination of the method), either in the spoiled or unspoiled cooked sausages. Conversely, the concentration of LAB varied depending on the product. In the spoiled samples, the LAB concentration ranged between 3 and 8 log CFU/g, while in unspoiled samples, it was less than 10 CFU/g. Among the various batches of spoiled cooked salami, only two species were detected: *Leuc. gelidum* and *Latilactobacillus sakei*. Specifically, out of the 600 identified colonies, 580 were identified as *Leuc. gelidum* and 20 as *Latilactobacillus sakei*. This strain was isolated only in the dilutions at the level of 10^−3^ CFU/g product, as demonstrated by the culture-independent technique (Table 3). *Leuc. gelidum* was also isolated using a combination of methods, including direct streaking of the yellowish patina from the spoiled salami onto the plate, decimal dilutions (up to 10^−8^ CFU/g), and the culture-independent method at each dilution (Table 3).

Sliced cooked meats prepacked in MAP can be subjected to microbial contamination and spoilage. Numerous studies on the microbiota of these products have consistently shown that lactic acid bacteria (LAB) are the primary contaminating microorganisms, multiplying throughout the shelf life and reaching impressively high numbers at 10^7^–10^9^ CFU/g [3,29]. Inducing microbial development, many factors such as temperature, pH, water activity (Aw > 0.96), nutrient availability, redox potential, and ATM composition have been found to affect food preservation along the cold chain, leading to waste and financial losses [15,30,31]. Because LAB produce hydrogen peroxide and organic acids, which have inherent antibacterial properties, they can help preserve meat. However, LAB can cause spoilage, including discoloration, changes in flavor, consistency, and odor, as well as the formation of films or slime, all of which can shorten the shelf life of cooked and MAP-packaged sausage [3,32]. The primary cause of this spoilage is heterofermenting bacteria such as *Leuconostoc carnosum*, *Leuc. gelidum*, *Carnobacterium divergens*, and *C. maltaromaticum*, or homofermenting LAB from the species *Latilactobacillus curvatus* and *L. sakei*. In addition, other strains can also grow. It appears that the growth of *Listeria* spp. and *Brochothrix thermosphacta* occurs when oxygen is present within the packages [3].

*L. sakei* is a common LAB found in meat, and it is utilized as a starter to ripen sausages and to provide bioprotection. It can, in fact, grow at psychrotrophic temperatures and undergo a significant amount of acidification. Since *Latilactobacillus sakei* is homolactic, it does not induce swelling in the sausages or in the packaging.

Despite being thermoduric, heat treatments such as pasteurization can lower its concentration. However, in regard to processed meats, if it survives, it may develop whitish patinas, which is occasionally observed in frankfurters stored for longer than their optimal shelf life.

In this instance, *L. sakei* cannot be held accountable for the spoilage even though it was there. It was detected up to a dilution of 10^−3^ CFU/g, which is obviously less than that of the *Leuc. gelidum* threshold. Moreover, the yellow hue observed on cooked sausages did not appear throughout its development in a model system based on meat extract. Consequently, only *Leuc. gelidum* must thus be regarded as the primary cause of the spoilage. Only colonies of this species were collected from the smears created, commencing with the yellow patina. The culture-independent methodology and the identification of colonies developed on high-dilution plates (>10^−4^ CFU/g) yielded identical results (Table 4). Moreover, the isolated strains of this species exhibited a yellowish patina resembling that of the cooked salami in a model system containing meat extract (Table 4).

*Leuconostoc* strains are psychrotrophic, heterofermentative, microaerophilic, belong to the LAB group, and produce spoilage [11,15,16]. In actuality, they generate a variety of compounds, including ethyl acetate, lactic acid, and acetic acid. If these substances are found in delicatessen products, they may serve as spoilage indicators [14,15]. Despite being thought of as thermoduric, *Leuconostoc* strains can be removed with heat treatments applied during the cooking process to produce cooked, cured meats. However, they are frequently identified in delicatessen items that are either filled or cooked in their entirety. *Leuc. carnosum* is one of the most commonly isolated species [1,6,13], followed by *Leuc. gelidum* and *Leuc. mesenteroides* [7]. Once these meats are cooked and finally pasteurized, the presence of these species is further highlighted [1]. Cooking, or heat treatment, usually renders most of the bacteria in the meat inactive. In fact, the microorganisms count in these kinds of cured meats is nearly invariably less than the method’s detection limit (<10 CFU/g). Only spore-forming bacteria and some LAB strains can survive at a temperature of 74 °C, which is sufficient to kill many other non-spore-forming bacteria. Additionally, a number of researchers have shown that cooked delicatessen products can still support LAB, Enterobacteriaceae, and other non-spore-forming Gram-negative bacteria, albeit at a decreased rate [1].

Indeed, sublethal harm to non-spore-forming microorganisms can occur when cooked at temperatures above 74 °C for longer than 10 min [33]. In addition, after heat treatment, injured cells may even revive and consequently normally develop [33]. Furthermore, it has been increasingly emphasized that the species that are most prevalent in cooked sausage are also present in raw meat after churning [34]. In fact, heat treatment used in the production of cured meats eliminates only 50% of bacteria, including *C. divergens*, *Latilactobacillus sakei* (former *Lactobacillus*), *Carnobacterium maltaromaticum*, *Leuc. carnosum*, *Leuc. gelidum*, *Leuc. mesenteroides*, and *Weissella* spp. [11,14,34].

The propensity of LAB to proliferate more quickly than other bacteria in ecosystems of cooked meats held in refrigeration, as well as after postcooking and packing recontamination, is the main reason for their presence in cooked, processed meats [35]. *Leuc. gelidum* and *L. sakei* can contaminate the product during the slicing and packaging stages, even in the case of the product under review, since the cooking temperature utilized is capable of eliminating these microbes (Table 1, Table 2, Table 3 and Table 4).

In contrast to what other authors have noted in their investigations of whole-piece cooked delicatessen products [11,36,37], the presence of LAB belonging to the *Carnobacterium* genus could not be proven. Since the product under consideration is made of minced pork, it is likely that the mincing process caused a rise in exudate, which, during heating, was supplemented with molecules beneficial to microbial activity; thus, a selection of LAB species was carried out. Moreover, grinding permits LAB to be distributed more widely throughout the meat. Despite analyzing whole-piece meat products, Veselá et al. [1] and Dušková et al. [7] were unable to isolate carnobacteria from prepared delicatessen products. In actuality, the lack of carnobacteria can result from these products being stored at temperatures below 12 °C [1]. It is known that *Leuconostoc* strains and *L. sakei* are more psychrotrophic than carnobacteria [11].

The activity of *Leuc. gelidum* that were isolated and chosen from the modified salami under investigation are shown in Table 4. It is evident that the isolated strains are able to grow at 4 °C with a GT at a level of approximately 15 ± 1 h, in addition to producing organic acids. Additionally, the isolated *L. sakei* strains exhibited a high degree of psychrotrophy. Their GT closely resembled (*p* > 0.05) the findings from the *Leuc gelidum* GT (16 ± 2 h).

The capacity of LAB to grow at refrigerated temperatures varies. These bacteria can be classified according to the temperature range in which they proliferate as true psychrotrophs or as cold-acclimated mesophiles [1,2]. For instance, mesophilic LAB that have adapted to low temperatures—the so-called induced psychrotrophs—such as *L. sakei*, *L. curvatus*, *Leuc. carnosum*, *Leuc. mesenteroides*, *Carnobacterium* spp., and *Weissella* spp.—belong to the first group. On the other hand, the strictly psychrophilic second group includes *L. fuchuensis*, *Leuc. gelidum*, and *Dellaglioa algida*, which are incapable of growing at 30 °C [12] but grow quickly at 6.5 and 15 °C, as reported by Veselá et al. [1].

Because producers want to give their products the longest shelf life possible to gain a competitive edge in the market, processed meat products, both sliced and unsliced, often have a shelf life of 21–28 days and, very rarely, 45 days.

The concentration and species of surviving microorganisms, as well as the storage temperature, have a significant impact on shelf life, as heat treatment does not sterilize the product. As our investigation has shown, the spoilage characterized by a yellow patina and indications of sourness was quickly caused by contamination of the product by psychrotrophic bacteria, which other researchers [1] thought to be psychrophilic.

### 3.2. Identification of Volatile Compounds of the Spoilage

The levels of ketones, carboxylic acid, and esters varied in the cooked salami samples. This feature is emphasized in Table 5, which lists only the components whose concentrations varied considerably between the spoiled and the unspoiled cooked sausages. It is evident that the volatile chemicals included alcohols, carboxylic acids, ketones, and esters. Since the concentrations of aldehydes did not differ between the spoiled and unspoiled samples, they are not reported. The compounds that remained unchanged included the following ketones: 2-propanone, 2-butanone, 2-pentanone, and 3-hydroxy-2-butanone.

The amount of ethanol in the spoiled samples was greater than that in the unspoiled sausages, and its concentration varied considerably (*p* < 0.05). Similarly, in the spoiled samples, the acetic acid concentration was noticeably greater. Finally, the unspoiled samples had an ethyl acetate concentration twice that of the spoiled ones [11,15]. Finally, the level of the ketones, except for 2-butanone, was higher in the unspoiled cooked sausages (*p* < 0.05). Among ketones, only the level of 2-butanone increased in the spoiled salami (*p*< 0.05).

The activity of *Leuc. gelidum*, which is heterofermentative and produces lactic acid as well as, more importantly, acetic acid and ethanol, was the cause of the notable variations in the volatilome. The combination of several chemicals results in the sensory profile. Olfactory deficiencies frequently result from an imbalance in the relative ratios of the molecules present rather than from the presence of a particular foreign molecule. Cured meats frequently contain molecules such as 2-butanone, but these molecules can only cause problems when present in large amounts [38]. They may have originated from bacteria that metabolized pyruvate [39,40]. In general, LAB, and specifically *Leuconostoc* strains, produce ketones. Specifically, they yield 2-butanone, which was shown to be more prevalent in the spoiled samples. The increased levels of ethanol and acetic acid in the spoiled samples can be taken into account in the same way. Both are typical outcomes of LAB heterolactic fermentation [40].

Based on these suppositions, the existence and function of *Leuc. gelidum* are adequate to account for the variations in these molecule concentrations between the spoiled and unspoiled cooked sausages.

### 3.3. Inhibitory Activity of Sodium Lactate, Sodium Diacetate, and Sodium Acetate

Table 6 displays information about the use of antimicrobial compounds against *Leuc. gelidum*. Sodium lactate (SL), sodium acetate (SA), and a combination of sodium lactate and sodium diacetate were the antimicrobial agents used. Thirty days of testing was conducted at two different temperatures: 4 °C for the entire test period or 10 days at 4 °C and the remaining 20 days at 8 °C. Given that this temperature may be representative of storage refrigerators and higher than the optimal temperature, it was utilized a thermal abuse temperature in this instance for two-thirds of the period.

SL was added at 1.5%, and SA was added at 0.1% or at 1.5% when mixed with 0.25% SDA. Increasing the concentrations of these antimicrobial agents can cause a change in the flavor. It is well known that SL has a bitter flavor, while SA has a sour taste.

Over the past 20 years, there has been a rise in the use of organic acid salt combinations, primarily sodium lactate (SL) with sodium acetate (SA) or sodium diacetate (SDA), for the purpose of controlling the growth of *Listeria monocytogenes* and spoilage microorganisms after they have been refrigerated in under vacuum (UV) or MAP frankfurters, sliced ham, and other cooked meats [41,42].

In the meat industry globally, various concentrations of SL, either alone or in combination with an SA or SDA, are currently approved and used as antilisteria agents [42]. These antimicrobial agents can also inhibit LAB and other non-LAB-spoiling bacteria in vitro (on nutrient agar) [42,43]. Numerous studies on the antilisterial effects of SL, SA, SDA, and other treatments with organic acid salts during the storage of different cooked meat products have been published in the scientific literature. These studies have shown that the spoilage microbiota, which is primarily composed of LAB, grows more slowly in their presence [41,42]. In fact, the antimicrobial agents employed in this study did not completely inhibit the spoiler growth inoculated at a level of 2 log CFU/mL. Most antimicrobial-treated samples showed decreased growth or a slowdown in growth compared with the control. The results from the combination treatment appeared superior to those from the other antimicrobial treatments used separately (*p* < 0.05). However, there was no discernible change between the samples treated with SL and those treated with SA (*p* > 0.05).

Nevertheless, there was still a significant difference (*p* < 0.05) between the outcomes achieved with these antimicrobial agents and the antimicrobial-free samples (control). These data are in agreement with those of Samelis and Kakouri [44], who emphasized that LAB are the primary spoiling agents of frankfurters treated with antimicrobial agents (SL, SL + SDA) and that these agents, irrespective of the temperature and concentration applied, minimize their growth in comparison to that of antimicrobial-free samples.

In fact, even though the change was especially noticeable in frankfurters with or without the addition of SL, antimicrobials had no effect on the growth of LAB at 15 days in the case of extreme thermal abuse (12 °C) [39]. In fact, a patina associated with LAB activity, such as *Latilactobacillus sakei/curvatus*, was observed in those samples. In our study, the patina, which was yellow in color and plainly visible by day 21, was caused by the purposeful or accidental contamination of *Leuc. gelidum*. However, Samelis and Kakouri [44] concluded that the antimicrobial agents used hindered the growth of lactobacilli and allied genera, favoring their dominance at the expense of *Leuconostoc* strains, which were difficult to grow in their samples.

*Leuc. gelidum* had a growth delay in our experiment depending on the temperature utilized and the presence of antimicrobial agents. However, the growth of the autochthonous LAB of the cooked sausages was not influenced by the antimicrobial agents, considering they reached values of 6 log CFU/g at the end of the storage.

Samples that were inoculated or not with *Leuc. gelidum* were acceptable for up to 7–12 days after the microorganism’s activity caused the emergence of yellowish patinas. On the other hand, *Lactobacillus* and related genera were found to be partially inhibited by SDA by Samnelis and Kakouri [44]. Per their results, the modification was detected at 60 days in the presence of SL and between 30 and 60 days in the control samples. *Leuconostoc* strains were more prevalent; this was especially true for frankfurters supplemented with a combination of SL and SDA. Here the impact of this mixture on *Leuc. gelidum* was not noted. Nonetheless, it is possible to speculate that the observed effect may vary depending on the substrate/ecosystem (cooked salami vs. frankfurters), the metabolism of the microorganisms involved, and most importantly, the absence of commercial combinations with additional antimicrobials [45]. Indeed, it has been shown that lactates, acetates, and other salts of organic acids have selective effects on LAB during the storage of cooked meat products [46,47]. In fact, these authors have previously shown that commercial mixtures of lactate and acetate salts induce selective pressure in situ against microorganisms that deteriorate meat, especially cooked meat. Since *Carnobacterium* spp., *Weissella* spp., and *Leuconostoc* spp. are more sensitive to acids—especially acetate—than the *Latilactobacillus sakei/curvatus* group in vitro, the presence of these acids has a greater inhibitory effect on their activity [48,49]. Despite these findings, in our work, *L. sakei* was isolated up to a concentration of 3 log CFU/g, *Leu. gelidum* was the only source of change in the spoiled cooked sausages under investigation (Table 3). This result is probably also related to initial significant *Leuconostoc* strain contamination, the lack of antimicrobial agents, and possible heat abuse during product storage in the production facility. In actuality, within the first 8 days of storage, deterioration (yellow slime) had already begun to occur. Previous observations of spoilage by *Leuconostoc* spp. in frankfurters, including bulging of the packages and colored slimes or films during storage at 4 °C, and particularly at 12 °C, were made by Samelis and Georgiadou [50]. Only when antimicrobials were present did *L. sakei/curvatus* predominate in frankfurters kept at 4 °C [44], most likely as a result of their resistance to these agents.

### 3.4. Antimicrobial Effect of Hop Extract

In this study, hop extract was also used to inhibit *Leuc. gelidum* growth both in vitro and in vivo. The first experiment was performed to determine the MIC of the hop extract. As shown in Table 7, the MIC was approximately 0.008 mg of CAE/mL. No effect was observed when ethanol was added without the hop extract added. Abram et al. [51] obtained better results using different hop extracts from Slovenia, Austria, Germany, and the Czech Republic. In particular, they found that the antimicrobial activity against Gram-positive *Staphylococcus aureus* was extraordinary for all hop cone extracts (MIC < 0.003 mg/mL), while it was moderate (MIC > 0.16 mg/mL) against Gram-negative *Escherichia coli* O157:H7. It can be hypothesized that the differences between our and Abram et al. [51] data depend on the type of hop and the microorganism strains.

Yamaguchi et al. [52] and Flesar et al. [53] obtained better results than our study, but they evaluated the effects of hop extract against an acne-causing strain of *St. aureus* and against the Gram-positive bacterium *Paenibacillus larvae*, respectively. Conversely, higher MICs were found for hop extract against different *Staphylococcus aureus* strains [54] and against Gram-negative *E. coli* O157:H7 (0.19 < MIC < 0.43 mg/mL). The effectiveness of these treatments may depend on the target strains.

Hop extract inhibition was also demonstrated in vivo (Table 8). The MIC at which different hop extract concentrations were added was approximately 4 mg/kg product (Figure 1b). This concentration did not allow *Leuc. gelidum* growth intentionally inoculated in cooked sausages for up to 30 days (which represents the end of the shelf life of the product) either at 4 °C or at 4–8 °C. At a minor concentration (3 mg/kg) of the hop extract, the inoculated strains were visible at 4 °C and 4–8 °C up to 20 ± 2 days and 16 ± 2 days, respectively (Table 8).

Usually, hop cones or hop extracts are added to beer to provide a bitter flavor and aroma, but both are recognized to also have antimicrobial activity [17]. β-Resin component mixtures (lupulones) have been reported to have greater antimicrobial activity than isoαresins (humulones) [17,18]. It is well known that hop bitter acids inhibit Gram-positive bacteria, including *Bacillus*, *Micrococcus*, *Staphylococcus*, and other bacteria [17,18,19]. Inhibitory activity has also been reported for certain fungi [20]. However, the majority of studies on the antimicrobial effects of hop extracts have been evaluated in culture media or in wort and beer but not in food. Previous studies have shown that the components of hop resins also have antimicrobial activity against *L. monocytogenes* in microbiological media and in some foods [50]. However, the activity of antimicrobials in vitro often does not accurately represent their efficacy in food. Larson et al. [17] showed that hop resin extracts can inhibit *L. monocytogenes* in media and in certain foods, such as coleslaw, whole milk, and cottage cheese, but not in Camembert cheese or minimally processed food, and hypothesized that the antimicrobial activity of hop extracts in food would increase with acidity and decrease with fat content.

To our knowledge, our data represent the first report of the antimicrobial activities of hop extracts against *Leuc. gelidum* either in vitro or in vivo; therefore, there are no published data available for comparative analysis, either alone or in combination with other agents.

### 3.5. Sensorial Analysis

The twenty nonprofessional subjects were unable to distinguish the two types of cooked sausages (with or without hop extract added). The triangle test methodology [27] demonstrated that the presence of hop extract at the MIC did not influence the odor or flavor of the cooked sausage. So, they established that there was no difference between the two samples. Considering that hop extract does not affect the sensorial quality of cooked salami, its use is proposed as an antimicrobial agent against *Leuc. gelidum*.

### 3.6. Origin of the Contamination and Spoilage Risk Elimination

Finally, since spoilage was found in a single production batch that was created in a single day, it was thought that the contamination caused by *Leuc. gelidum* or *L. sakei* was natural and stems from the environment. It can be specifically theorized that a product cut the previous day polluted the environment and, in particular, the slicing lines. Consequently, due to inadequate cleaning of the slicing lines, the contamination spread to the cooked salami under study.

It is impossible that the contamination originates from raw meat because, as demonstrated, both *Leuc. gelidum* and *L. sakei* were eliminated by cooking, as also shown in the in vitro tests and by the analysis of the unspoiled cooked sausages. Consequently, it is highly unlikely that these microbes could originate from the raw meat or the phases before cooking. In addition, the level of microorganisms present after the cooking process in unspoiled cooked sausages was less than 10 CFU/g product, which is the lower limit of determination of the method. Thus, the spoilage was caused by environmental contamination of the slicing lines. It can be hypothesized that the slicing lines were not sufficiently sanitized, and this has allowed the contamination of the investigated samples.

Indeed, at the end of the spoiled batch, an adequate disinfection of the slicing lines eliminated the risk of subsequent contamination and spoilage. Indeed, subsequent lots of production were not spoiled, despite them being part of the same batch of meat of the spoiled sausages.

Thus, it can be inferred that the risk of spoilage can be eliminated or at least reduced by strictly implementing a HACCP system and preoperational procedures (environmental and equipment sanitization). This system proposes high-quality raw materials, suitable technology chosen based on the selection of suitable cooking times and temperatures, hygienic equipment and surroundings, and the removal of contaminants during the slicing and packaging processes.

## 4. Conclusions

Sliced cooked sausage packaged in a modified atmosphere is a popular ready-to-eat product subjected to abundant microbial contamination throughout its shelf life that can lead to deterioration of both its sensorial properties and safety. Lactic acid bacteria, particularly *Leuconostoc* spp., can be the main spoilers of ready-to-eat meat products, originating from improper cooking, sanitization practices, and recontamination during slicing and packaging. In this study, the presence of *Leuc. gelidum* led to the formation of a yellow patina on the cooked sausages. Although *L. sakei* was also present, it did not contribute to the yellow patina. The spoilage activity of *Leuc. gelidum* was further confirmed by volatilome. Indeed, this analysis revealed higher concentrations of lactic acid, acetic acid, ethanol, and ethyl acetate in spoiled products compared with unspoiled ones, attributable to the activity of this heterofermentative bacterium. Various traditional antimicrobial compounds were tested in model systems composed of cooked sausage extract to inhibit *Leuc. gelidum*. Sodium lactate, sodium acetate, and a combination of sodium lactate and sodium diacetate were used, but the data showed that these antimicrobial agents could only slow the growth of the spoilage bacteria. The scientific literature suggested the use of hop extract. The results demonstrated that hop extract could completely inhibit *Leuc. gelidum*, showing a minimal inhibitory concentration (MIC) of approximately 0.008 mg CAE/mL in synthetic broth and 4 mg CAE/kg in cooked sausage slices. Based on the sensorial analysis, the addition of hop extract at the MIC did not change the odor or the flavor of the cooked sausages. Considering the results on the effect of hop extract, further studies at different levels should be suggested. In particular, the effect of different hop genotypes, coming from various regions, against spoilage microorganisms should be investigated. Additionally, future studies should focus on dehydrating the ethanolic hop extract to produce a powder that can be easily stored and used in the food industry. The studies will include the effect on different foods, such as meat and meat products, cheeses, and vegetables. In addition, the potential antibacterial effects demonstrated in our study could also be applied in the pharmaceutical, veterinary, and cosmetic industries.

## Figures and Tables

**Table 1:** Phases of the cooked sausage production.

Phase	Temperature/Time
Raw meat and fat	4 ± 2 °C
Trimming/grinding	12 °C
Addition of tanning/kneading	7 °C
Rest	2–4 °C—12 h
Casing	12 °C
Smoking	66 °C—24 h
Cooking	72–78 °C—6 h
Cooling/slicing	2–4 °C
MAP/Storing	4 ± 2 °C

Notes. MAP, modified atmosphere 20% CO_2_, 70% N_2_, <0.2% O_2._

**Table 2:** Antimicrobials and their concentrations added in model system (cooked salami).

Antimicrobial Compound	Concentration	Number Samples/Temperature	
		4 °C	4–8 °C
Sodium lactate	1.5%	15	15
Sodium acetate	0.1%	15	15
Sodium lactate/sodium diacetate	1.5/0.25%	15	15
Control		15	15

Notes. Storage stored at 4 °C for up to 30 days and at 4 °C for 10 days, then at 8 °C for 20 days.

**Table 3:** Isolated strains at different dilutions.

Microorganism	Accession Number	Serial Dilutions					
		10^−3^	10^−4^	10^−5^	10^−6^	10^−7^	10^−8^
*Leuconostoc gelidum*	MK948921.1	+	+	+	+	+	+
*Latilactobacillus sakei*	CP113247.1	+	-	-	-	-	-

Notes. Data: CFU/mL; + presence; - no presence

**Table 4:** Physico-chemical characters of the strains isolated from cooked sausage.

Microorganism	Yellow Slime	pH	Pasteurization 72 °C for 5 min	** Growth at 4 °C
*Leuconostoc gelidum*	+	5.5 ± 0.2 a *	−6 log CFU/g	15 ± 1 a *
*Latilactobacillus sakei*	-	5.3 ± 0.2 a *	−6 log CFU/g	16 ± 2 a *

Notes. +/- positive/negative production of yellow slime on meat or meat extract medium; ** average generation time in h: * data represent the means ± the standard deviations of all samples. The means with the same letters within the columns are not significantly different (*p* < 0.05).

**Table 5:** Volatile compounds in unspoiled and spoiled cooked sausages.

RT	Compound	RI	Unspoiled		Spoiled	
			Mean	(±) SD	Mean	(±) SD
	Ketones					
2.29	2-Propanone	819	2.51	0.02 a	1.65	0.08 b
3.17	2-Butanone	907	6.04	0.03 b	9.33	0.01 a
4.72	2-Pentanone	961	8.45	0.07 a	0.28	0.01 b
17.63	3-Hydroxy-2-butanone	1284	37.35	0.64 a	5.80	0.19 b
	Alcohols					
3.87	Ethanol	932	16.90	0.08 b	26.11	0.02 a
	Carbossilic acid					
22.23	Acetic acid	1449	5.48	0.11 b	7.51	0.21 a
27.04	3-Methylbutanoic acid	1666	1.88	0.07 a	1.04	0.02 b
	Esters					
3.05	Ethyl acetate	888	2.26	0.04 a	0.88	0.07 b

Notes. Average of 10 samples expressed in µg/kg: RT, retention time; RI, retention index; SD, standard deviation. Data represent means ± the standard deviations of all samples. Means with the same letter following the lines are not significantly different (*p* < 0.05).

**Table 6:** Dynamic of *Leuconosatoc gelidum* in model system stored at 4 °C and 4–8 °C up to 30 days.

Temperature	Treatment	Days				
		0	5	10	20	30
4 °C	C	2.1 ± 0.2 a	2.1 ± 0.2 a	5.4 ± 0.3 a	6.7 ± 0.2 b	8.4 ± 0.1 b
	SL 1.5%	2.0 ± 0.2 a	2.0 ± 0.3 a	5.4 ± 0.1 a	6.3 ± 0.2 a	7.9 ± 0.2 a
	SA 1.5%	2.1 ± 0.2 a	2.0 ± 0.1 a	5.2 ± 0.2 a	6.3 ± 0.1 a	7.9 ± 0.2 a
	SL 1.5% + SDA 0.25%	2.0 ± 0.2 a	2.1 ± 0.1 a	5.1 ± 0.2 a	6.0 ± 0.2 a	7.7 ± 0.3 c
4–8 °C	C	2.0 ± 0.1 a	2.0 ± 0.1 a	5.4 ± 0.1 a	7.2 ± 0.2 b	9.2 ± 0.2 b
	SL 1.5%	2.1 ± 0.2 a	2.0 ± 0.1 a	5.3 ± 0.2 a	6.9 ± 0.2 a	8.6 ± 0.1 c
	SA 1.5%	2.0 ± 0.2 a	2.0 ± 0.3 a	5.3 ± 0.2 a	6.9 ± 0.3 a	8.7 ± 0.1 c
	SL 1.5% + SDA 0.25%	2.0 ± 0.2 a	2.0 ± 0.2 a	5.2 ± 0.1 a	6.7 ± 0.2 a	8.2 ± 0.2 b

Notes. Average data ± standard deviation, CFU/mL; 4–8 °C, incubation for 10 days at 4 °C and 20 days at 8 °C (heat abuse). The averages with the same letter following the columns are not significantly different (*p* < 0.05). SL, sodium lactate; SDA, sodium diacetate; SA, sodium acetate.

**Table 7:** Antimicrobial hop extract (MIC evaluation).

Hop Extract Concentration mg/mL	Strains		
	1	2	3
0.08	-	-	-
0.04	-	-	-
0.016	-	-	-
0.008	-	-	-
0.004	+	+	+

Notes. + growth; - no growth.

**Table 8:** Behavior of *Leuc. gelidum* growth in cooked sausage in modified atmosphere packaged and stored at 4 °C up to 30 days and at 4 °C for 10 days, then at 8 °C for 20 days.

Hop Extract Concentration (mg/kg)	Day of Visible Growth	
	4 °C	4–8 °C
0	8 ± 1	8 ± 1
2	15 ± 2	13 ± 2
3	20 ± 2	16 ± 2
4	-	-
5	-	-

Notes. - No growth till the end of the shelf life (30 days); average data ± standard deviation, CFU/mL; 4–8 °C, incubation for 10 days at 4 °C and 20 days at 8 °C (heat abuse).

## Data Availability

Data are contained within the article.

## References

[1] Veselá H., Dorotíková K., Dušková M., Furmančíková P., Šedo O., Kameník J. (2022). The Pork Meat or the Environment of the Production Facility? The Effect of Individual Technological Steps on the Bacterial Contamination in Cooked Hams. Microorganisms.

[2] Kameník J., Bogdanovičová K., Dorotíková K. (2019). Haltbarkeit von Geschnittenem Kochschinken in Modifizierter Atmosphäre. Fleischwirtschaft.

[3] Raimondi S., Luciani R., Sirangelo T.M., Amaretti A., Leonardi A., Ulrici A., Foca G., D’Auria G., Moya A., Zuliani V. (2019). Microbiota of Sliced Cooked Ham Packaged in Modified Atmosphere throughout the Shelf Life: Microbiota of Sliced Cooked Ham in MAP. Int. J. Food Microbiol..

[4] Mataragas M., Drosinos E.H., Vaidanis A., Metaxopoulos I. (2006). Development of a Predictive Model for Spoilage of Cooked Cured Meat Products and Its Validation under Constant and Dynamic Temperature Storage Conditions. J. Food Sci..

[5] Pothakos V., Snauwaert C., De Vos P., Huys G., Devlieghere F. (2014). Psychrotrophic Members of *Leuconostoc gasicomitatum, Leuconostoc gelidum* and *Lactococcus piscium* Dominate at the End of Shelf-Life in Packaged and Chilled-Stored Food Products in Belgium. Food Microbiol..

[6] Bjorkroth K.J., Geisen R., Schillinger U., Weiss N., De Vos P., Holzapfel W.H., Korkeala H.J., Vandamme P. (2000). Characterization of *Leuconostoc gasicomitatum* Sp. Nov., Associated with Spoiled Raw Tomato-Marinated Broiler Meat Strips Packaged under Modified-Atmosphere Conditions. Appl. Environ. Microbiol..

[7] Dušková M., Kameník J., Lačanin I., Šedo O., Zdráhal Z. (2016). Lactic Acid Bacteria in Cooked Hams—Sources of Contamination and Chances of Survival in the Product. Food Control.

[8] Pothakos V., Samapundo S., Devlieghere F. (2012). Total Mesophilic Counts Underestimate in Many Cases the Contamination Levels of Psychrotrophic Lactic Acid Bacteria (LAB) in Chilled-Stored Food Products at the End of Their Shelf-Life. Food Microbiol..

[9] Björkroth K.J., Korkeala H.J. (1997). Use of RRNA Gene Restriction Patterns to Evaluate Lactic Acid Bacterium Contamination of Vacuum-Packaged Sliced Cooked Whole-Meat Product in a Meat Processing Plant. Appl. Environ. Microbiol..

[10] Doulgeraki A.I., Paramithiotis S., Kagkli D.M., Nychas G.J.E. (2010). Lactic Acid Bacteria Population Dynamics during Minced Beef Storage under Aerobic or Modified Atmosphere Packaging Conditions. Food Microbiol..

[11] Comi G., Iacumin L. (2012). Identification and Process Origin of Bacteria Responsible for Cavities and Volatile Off-Flavour Compounds in Artisan Cooked Ham. Int. J. Food Sci. Technol..

[12] Lyhs U., Koort J.M.K., Lundström H.S., Björkroth K.J. (2004). *Leuconostoc gelidum* and *Leuconostoc gasicomitatum* Strains Dominated the Lactic Acid Bacterium Population Associated with Strong Slime Formation in an Acetic-Acid Herring Preserve. Int. J. Food Microbiol..

[13] Vihavainen E.J., Björkroth K.J. (2007). Spoilage of Value-Added, High-Oxygen Modified-Atmosphere Packaged Raw Beef Steaks by *Leuconostoc gasicomitatum* and *Leuconostoc gelidum*. Int. J. Food Microbiol..

[14] Cantoni C., Milesi S., Iacumin L., Comi G. (2009). *Leuconostoc* e Rigonfiamento in Prodotti Alimentari. Ind. Aliment..

[15] Comi G., Andyanto D., Manzano M., Iacumin L. (2016). *Lactococcus lactis* and *Lactobacillus sakei* as Bio-Protective Culture to Eliminate *Leuconostoc mesenteroides* Spoilage and Improve the Shelf Life and Sensorial Characteristics of Commercial Cooked Bacon. Food Microbiol..

[16] Iacumin L., Cecchini F., Manzano M., Osualdini M., Boscolo D., Orlic S., Comi G. (2009). Description of the Microflora of Sourdoughs by Culture-Dependent and Culture-Independent Methods. Food Microbiol..

[17] Larson A.E., Yu R.R.Y., Lee O.A., Price S., Haas G.J., Johnson E.A. (1996). Antimicrobial Activity of Hop Extracts against *Listeria monocytogenes* in Media and in Food. Int. J. Food Microbiol..

[18] Hough J.S., Howard G.A., Slater C.A. (1957). Bacteriostatic Activities of Hop Resin Materials. J. Inst. Brew..

[19] Schmalreck A.F., Teuber M., Reininger W., Hartl A. (1975). Structural Features Determining the Antibiotic Potencies of Natural and Synthetic Hop Bitter Resins, Their Precursors and Derivatives. Can. J. Microbiol..

[20] Mizobuchi S., Sato Y. (1985). Antifungal Activities of Hop Bitter Resins and Related Compounds. Agric. Biol. Chem..

[21] Cocolin L., Manzano M., Cantoni C., Comi G. (2001). Denaturing Gradient Gel Electrophoresis Analysis of the 16S RRNA Gene V1 Region to Monitor Dynamic Changes in the Bacterial Population during Fermentation of Italian Sausages. Appl. Environ. Microbiol..

[22] Rantsiou K., Urso R., Iacumin L., Cantoni C., Cattaneo P., Comi G., Cocolin L. (2005). Culture-Dependent and -Independent Methods to Investigate the Microbial Ecology of Italian Fermented Sausages. Appl. Environ. Microbiol..

[23] Altschul S.F., Madden T.L., Schäffer A.A., Zhang J., Zhang Z., Miller W., Lipman D.J. (1997). Gapped BLAST and PSI-BLAST: A New Generation of Protein Database Search Programs. Nucleic Acids Res..

[24] Chiesa L., Duchini M., Iacumin L., Boscolo D., Comi G., Cantoni C. (2006). Caratteristiche Chimiche e Batteriologiche Di Salami Della Lomellina. Arch. Vet. Ital..

[25] Stout M.J., Brovont R.A., Duffey S.S. (1998). Effect of Nitrogen Avilability on Expression of Constitutive and Inducible Chemical Defenses in Tomato, *Lycopersicon esculentum*. J. Chem. Ecol..

[26] Singleton V.L., Rossi J.A. (1965). Colorimetry of Total Phenolics with Phosphomolybdic-Phosphotungstic Acid Reagents. Am. J. Enol. Vitic..

[27] ISO 4120:2004. Triangle Test Methodology. Standard Test Method for Sensory Analysis—General Guidance for the Design of test Rooms.

[28] Stone H., Sidel J.L. (2004). Sensory Evaluation Practices.

[29] Menezes N.M.C., Martins W.F., Longhi D.A., de Aragão G.M.F. (2018). Modeling the Effect of Oregano Essential Oil on Shelf-Life Extension of Vacuum-Packed Cooked Sliced Ham. Meat Sci..

[30] Iulietto M.F., Sechi P., Borgogni E., Cenci-Goga B.T. (2015). Meat Spoilage: A Critical Review of a Neglected Alteration Due to Ropy Slime Producing Bacteria. Ital. J. Anim. Sci..

[31] Spampinato G., Candeliere F., Amaretti A., Licciardello F., Rossi M., Raimondi S. (2022). Microbiota Survey of Sliced Cooked Ham During the Secondary Shelf Life. Front. Microbiol..

[32] Kreyenschmidt J., Hübner A., Beierle E., Chonsch L., Scherer A., Petersen B. (2010). Determination of the Shelf Life of Sliced Cooked Ham Based on the Growth of Lactic Acid Bacteria in Different Steps of the Chain. J. Appl. Microbiol..

[33] Wu V.C.H. (2008). A Review of Microbial Injury and Recovery Methods in Food. Food Microbiol..

[34] Zagdoun M., Coeuret G., N’Dione M., Champomier-Vergès M.C., Chaillou S. (2020). Large Microbiota Survey Reveals How the Microbial Ecology of Cooked Ham Is Shaped by Different Processing Steps. Food Microbiol..

[35] Vasilopoulos C., De Maere H., De Mey E., Paelinck H., De Vuyst L., Leroy F. (2010). Technology-Induced Selection towards the Spoilage Microbiota of Artisan-Type Cooked Ham Packed under Modified Atmosphere. Food Microbiol..

[36] Pothakos V., Snauwaert C., De Vos P., Huys G., Devlieghere F. (2014). Monitoring Psychrotrophic Lactic Acid Bacteria Contamination in a Ready-to-Eat Vegetable Salad Production Environment. Int. J. Food Microbiol..

[37] Audenaert K., D’Haene K., Messens K., Ruyssen T., Vandamme P., Huys G. (2010). Diversity of Lactic Acid Bacteria from Modified Atmosphere Packaged Sliced Cooked Meat Products at Sell-by Date Assessed by PCR-Denaturing Gradient Gel Electrophoresis. Food Microbiol..

[38] Tabanelli G., Montanari C., Grazia L., Lanciotti R., Gardini F. (2013). Effects of Aw at Packaging Time and Atmosphere Composition on Aroma Profile, Biogenic Amine Content and Microbiological Features of Dry Fermented Sausages. Meat Sci..

[39] Carballo J., Mehta B.M., Kamal-Eldin A., Iwanski R.Z. (2012). The Role of Fermentation Reactions in the Generation of Flavor and Aroma of Foods. Fermentation: Effects on Food Properties.

[40] Montanari C., Barbieri F., Gardini G., Magnani R., Gottardi D., Gardini F., Tabanelli G. (2022). Effects of Starter Cultures and Type of Casings on the Microbial Features and Volatile Profile of Fermented Sausages. Fermentation.

[41] Geornaras I., Skandamis P.N., Belk K.E., Scanga J.A., Kendall P.A., Smith G.C., Sofos J.N. (2006). Postprocess Control of *Listeria monocytogenes* on Commercial Frankfurters Formulated with and without Antimicrobials and Stored at 10 °C. J. Food Prot..

[42] Brasileiro I.S., Barbosa M., Igarashi M.C., Biscola V., Maffei D.F., Landgraf M., Franco B.D.G. (2016). de M. Use of Growth Inhibitors for Control of *Listeria monocytogenes* in Heat-Processed Ready-to-Eat Meat Products Simulating Post-Processing Contamination. LWT.

[43] Horita C.N., Baptista R.C., Caturla M.Y.R., Lorenzo J.M., Barba F.J., Sant’Ana A.S. (2018). Combining Reformulation, Active Packaging and Non-Thermal Post-Packaging Decontamination Technologies to Increase the Microbiological Quality and Safety of Cooked Ready-to-Eat Meat Products. Trends Food Sci. Technol..

[44] Samelis J., Kakouri A. (2021). Growth Inhibitory and Selective Pressure Effects of Sodium Diacetate on the Spoilage Microbiota of Frankfurters Stored at 4 °C and 12 °C in Vacuum. Foods.

[45] Andreevskaya M., Jääskeläinen E., Johansson P., Ylinen A., Paulin L., Björkroth J., Auvinen P. (2018). Food Spoilage-Associated *Leuconostoc, Lactococcus*, and *Lactobacillus* Species Display Different Survival Strategies in Response to Competition. Appl. Environ. Microbiol..

[46] Ibrahim S.A., Ayivi R.D., Zimmerman T., Siddiqui S.A., Altemimi A.B., Fidan H., Esatbeyoglu T., Bakhshayesh R.V. (2021). Lactic Acid Bacteria as Antimicrobial Agents: Food Safety and Microbial Food Spoilage Prevention. Foods.

[47] Drosinos E.H., Mataragas M., Kampani A., Kritikos D., Metaxopoulos I. (2006). Inhibitory Effect of Organic Acid Salts on Spoilage Flora in Culture Medium and Cured Cooked Meat Products under Commercial Manufacturing Conditions. Meat Sci..

[48] Bouju-Albert A., Pilet M.F., Guillou S. (2018). Influence of Lactate and Acetate Removal on the Microbiota of French Fresh Pork Sausages. Food Microbiol..

[49] Peirson M.D., Guan T.Y., Holley R.A. (2003). Thermal Resistances and Lactate and Diacetate Sensitivities of Bacteria Causing Bologna Discolouration. Int. J. Food Microbiol..

[50] Samelis J., Georgiadou K.G. (2000). The Microbial Association of Greek Taverna Sausage Stored at 4 and 10 °C in Air, Vacuum or 100% Carbon Dioxide, and Its Spoilage Potential. J. Appl. Microbiol..

[51] Abram V., Čeh B., Vidmar M., Hercezi M., Lazić N., Bucik V., Možina S.S., Košir I.J., Kač M., Demšar L. (2015). A Comparison of Antioxidant and Antimicrobial Activity between Hop Leaves and Hop Cones. Ind. Crops Prod..

[52] Yamaguchi N., Satoh-Yamaguchi K., Ono M. (2009). In Vitro Evaluation of Antibacterial, Anticollagenase, and Antioxidant Activities of Hop Components (*Humulus Lupulus*) Addressing *Acne vulgaris*. Phytomedicine.

[53] Flesar J., Havlik J., Kloucek P., Rada V., Titera D., Bednar M., Stropnicky M., Kokoska L. (2010). In Vitro Growth-Inhibitory Effect of Plant-Derived Extracts and Compounds against *Paenibacillus larvae* and Their Acute Oral Toxicity to Adult Honey Bees. Vet. Microbiol..

[54] Rozalski M., Micota B., Sadowska B., Stochmal A., Jedrejek D., Wieckowska-Szakiel M., Rozalska B. (2013). Antiadherent and Antibiofilm Activity of *Humulus lupulus* L. Derived Products: New Pharmacological Properties. Biomed Res. Int..

